# Machine Learning for Short-Term Mortality in Acute Decompensation of Liver Cirrhosis: Better than MELD Score

**DOI:** 10.3390/diagnostics14100981

**Published:** 2024-05-08

**Authors:** Nermin Salkić, Predrag Jovanović, Mislav Barišić Jaman, Nedim Selimović, Frane Paštrović, Ivica Grgurević

**Affiliations:** 1 Department of Internal Medicine, School of Medicine, University of Tuzla, 75000 Tuzla, Bosnia and Herzegovina; prredo@yahoo.com; 2 Department of Gastroenterology and Hepatology, University Clinical Center Tuzla, 75000 Tuzla, Bosnia and Herzegovina; nedim.selimovic95@gmail.com; 3 Department for Gastroenterology, Hepatology and Clinical Nutrition, School of Medicine, University of Zagreb, University Hospital Dubrava, 10000 Zagreb, Croatia; mislav.barisic.jaman@gmail.com (M.B.J.);; 4 Faculty of Pharmacy and Biochemistry, University of Zagreb, 10000 Zagreb, Croatia

**Keywords:** machine learning, mortality prediction, liver cirrhosis, MELD-Na score, MELD 3.0 score, liver transplantation, transplant selection

## Abstract

Prediction of short-term mortality in patients with acute decompensation of liver cirrhosis could be improved. We aimed to develop and validate two machine learning (ML) models for predicting 28-day and 90-day mortality in patients hospitalized with acute decompensated liver cirrhosis. We trained two artificial neural network (ANN)-based ML models using a training sample of 165 out of 290 (56.9%) patients, and then tested their predictive performance against Model of End-stage Liver Disease-Sodium (MELD-Na) and MELD 3.0 scores using a different validation sample of 125 out of 290 (43.1%) patients. The area under the ROC curve (AUC) for predicting 28-day mortality for the ML model was 0.811 (95%CI: 0.714- 0.907; *p* < 0.001), while the AUC for the MELD-Na score was 0.577 (95%CI: 0.435–0.720; *p* = 0.226) and for MELD 3.0 was 0.600 (95%CI: 0.462–0.739; *p* = 0.117). The area under the ROC curve (AUC) for predicting 90-day mortality for the ML model was 0.839 (95%CI: 0.776- 0.884; *p* < 0.001), while the AUC for the MELD-Na score was 0.682 (95%CI: 0.575–0.790; *p* = 0.002) and for MELD 3.0 was 0.703 (95%CI: 0.590–0.816; *p* < 0.001). Our study demonstrates that ML-based models for predicting short-term mortality in patients with acute decompensation of liver cirrhosis perform significantly better than MELD-Na and MELD 3.0 scores in a validation cohort.

## 1. Introduction

Liver cirrhosis is a chronic and progressive liver disease that is a leading cause of morbidity and mortality worldwide. In recent years, the prevalence of liver cirrhosis has been increasing due to the rising prevalence of obesity and associated non-alcoholic fatty liver disease, and alcoholism, despite the decreasing global burden of hepatitis B virus and hepatitis C virus-associated cirrhosis [1]. Decompensated liver cirrhosis is a clinical-stage disease characterized by the onset of complications such as ascites, hepatic encephalopathy, and variceal bleeding resulting from severely impaired liver function and/or high portal pressure, leading to inevitable death [2,3].

The prognosis of decompensated liver cirrhosis is poor with a high mortality rate, particularly in patients with acute decompensation. The average survival without liver transplantation is approximately two years [4]. Prediction of mortality in these patients is crucial for their management, including prioritization for liver transplantation, which is of special importance in the context of global organ shortage [5,6]. Several clinical scoring systems, such as the Child-Pugh score (CP score) and Model for End-Stage Liver Disease (MELD) score and its derivatives, such as MELD-Na and the recently introduced MELD 3.0 score, have been developed and used to predict mortality in patients with liver cirrhosis [7,8,9,10]. The MELD score and MELD-Na score are used as the standard scoring systems for organ allocation worldwide [11]. However, these scoring systems have limitations and leave room for improvement, especially for predicting short-term mortality [12,13].

Machine learning (ML) algorithms are increasingly utilized in all areas of human knowledge, including medicine, mainly due to their inherent ability to learn and improve over time and offer more accurate predictions, regardless of the outcome needed [14,15]. Hepatology is no exception, and ML has recently been explored to predict mortality in patients with decompensated liver cirrhosis [16,17,18,19,20,21,22]. These algorithms can analyze large and complex datasets, including clinical, laboratory, and radiological parameters, and identify patterns and associations that are not apparent through traditional statistical methods. Furthermore, ML algorithms can also be trained and validated using electronic health records and clinical databases and applied afterward in clinical settings for predicting outcomes based on newly acquired input data [23,24].

The development and validation of accurate ML models for predicting short-term mortality in patients with decompensated liver cirrhosis could have significant clinical implications. These models could help clinicians identify patients at high risk of mortality and provide them with more intensive and personalized treatment, as well as timely and life-saving interventions such as liver transplantation. Furthermore, when compared with traditionally used models such as MELD or CP score, these models could improve patient outcomes by facilitating earlier interventions, reducing healthcare costs, and enabling more efficient use of healthcare resources and organs available for transplantation.

Therefore, in this study, we aimed to develop and validate ML models for predicting 28-day and 90-day mortality in patients hospitalized with decompensated liver cirrhosis, regardless of the etiology of liver disease, and compare their predictive ability against MELD-Na and MELD 3.0 scores.

## 2. Materials and Methods

### 2.1. Patients

**Figure 1:**
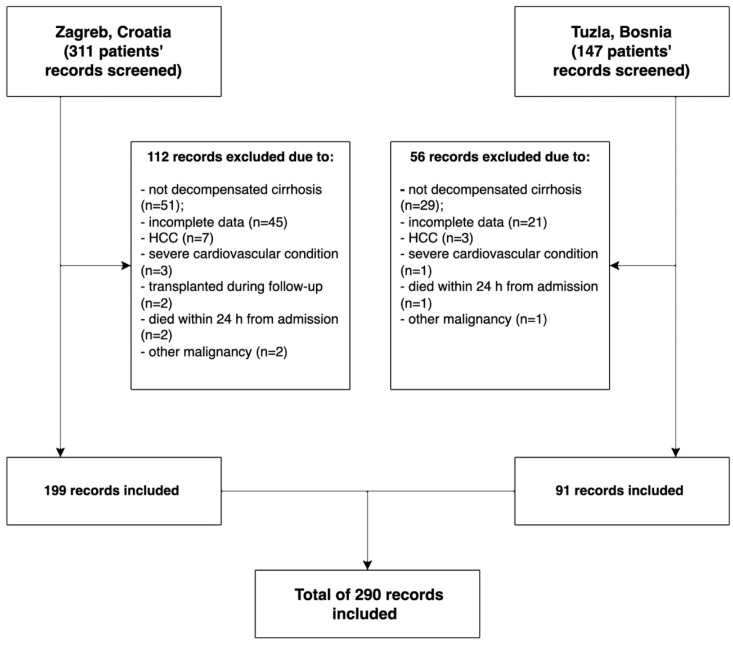
Flow diagram illustrating the selection of patient records.

In this study, we conducted a retrospective analysis of medical records from 290 patients who were hospitalized with decompensated liver cirrhosis at two gastroenterology departments in Croatia, Bosnia, and Herzegovina. Of these patients, 199 (68.6%) were from University Hospital Dubrava in Zagreb (between 2014–2017), while 91 (31.4%) were from University Clinical Center Tuzla (between 2016–2017)—Figure 1.

Inclusion criteria required the presence of acute decompensation of liver cirrhosis upon admission and available data on 28- and 90-day survival. Diagnosis of liver cirrhosis was established by liver biopsy (in most cases before decompensation) or unequivocal clinical criteria (including typical imaging of endoscopic and laboratory features) at the time of presentation, whereas the etiology of liver cirrhosis relied on the anamnestic data about harmful alcohol consumption, presence of features of metabolic syndrome, or confirmed by the biochemical, serological, or genetic tests typical for viral, autoimmune, or hereditary liver diseases. Acute decompensation was defined by the presence of overt ascites, encephalopathy, porto-hypertensive bleeding, or severe infection that required in-hospital treatment [25].

Patients with incomplete data, hepatocellular carcinoma, any other malignancy, severe cardiovascular conditions, those who died within 24 h of initial evaluation, transplanted patients within the follow-up period, and pediatric patients (age < 18 years) were excluded from the study. The study was approved by the Ethics Committees of both institutions.

### 2.2. Data

We collected clinical and biochemical parameters obtained on initial evaluation, including age, gender, etiology of liver disease, leukocyte and platelet count, serum levels of hemoglobin, creatinine, total bilirubin, sodium, albumin, and international normalized ratio (INR).

We also recorded the grade of ascites according to the Child-Pugh classification (grade 0—none; grade 1—treatment controlled; grade 2—not controlled on treatment) [10] and encephalopathy according to the West Haven Scale [26]. We calculated MELD-Na and MELD 3.0 scores for all patients on initial evaluation according to previously published formulas [8,9]. All of these variables were used as input variables for our ML models. We also recorded mortality after 28 and 90 days from initial evaluation, which were converted into dichotomous yes/no variables as output variables for our ML models.

### 2.3. Development of ML Model

We developed an ML model based on artificial neural network modeling, specifically using a Multilayer Perceptron Model within the Neural Network function in the SPSS 26.0 (SPSS, Chicago, IL, USA) software package to construct and train two separate models—one for predicting 28-day mortality and the other for predicting 90-day mortality.

To ensure the accuracy of the ANN, we trained the model using a process in which we added input data (patient data) and known output information (correct classification—in our case, mortality after 28 or 90 days) to each training sample. After each pair of input-output data passed through the ANN, the computed network output result was compared against the real outcome and the error rate was calculated. The weight of each connection was then adjusted based on the error rate, reducing the error between the real outcome and the actual output. This adjustment algorithm moves from layer to layer in the direction from output to input layer, called a back-propagation algorithm, which is the most widely used method for training ANNs [27].

The division of data into training and validation sets was carried out using a stratified random sampling method to ensure that both datasets were representative of the overall population. This stratification was done based on the outcome variable—the presence of acute decompensation events—to ensure that the proportion of outcomes was similar across both datasets, thereby reducing sample bias and improving the external validity of the model’s performance. This resulted in the sample being divided into two parts: 165/290 (56.9%) patients were used for training both models, while the remaining 125/290 (43.1%) were used for validating both models. We used the training sample to separately train both models, while the validation sample was used to assess their accuracies. The error for this sample gives an honest estimate of the predictive ability of both models since the validation cases were not used to build and train it.

To construct our ML models, we employed a back-propagation algorithm with automatic selection of the optimal network architecture, which was implemented with multiple strategies to mitigate overfitting and ensure the generalizability of our machine learning models. We utilized L2 regularization to penalize overly complex models by adding a magnitude-based penalty to the loss function. Additionally, we employed pruning to reduce model complexity by systematically removing less significant weights. The process of early stopping was employed to halt training when no improvements were observed on the validation set over a set number of epochs. Additionally, we utilized SPSS’s automatic feature for selecting the optimal network architecture, with the objective of optimizing both model complexity and performance based on validation data outcomes. We saved the predicted probabilities and network classification output for each patient and evaluated them in subsequent statistical analyses.

### 2.4. Statistical Analysis

All statistical analyses were performed using the SPSS 26.0 software package (SPSS, Chicago, IL, USA). Prior to analysis, all variables were subjected to a Kolmogorov-Smirnov test to ascertain their normal distribution. Descriptive statistics were used to determine the baseline characteristics of all variables. When appropriate, Student’s *t*-test with correction for unequal variances was used to compare quantitative variables, while the chi-square test was used for categorical variables. The diagnostic accuracy of both predictive models was evaluated using Receiver Operating Characteristics (ROC) analysis, and areas under the ROC curve (AUC) (which corresponds to c-statistics) were compared pairwise between the ML models and the MELD score. All tests were conducted at a 95% confidence level (*p* < 0.05).

## 3. Results

### 3.1. Baseline Characteristics

Table 1 presents the baseline characteristics of the 290 patients included in the study. The patients had a mean age of 63 (SD 10) years, ranging from 31 to 88 years. Of the patients, 211/290 (72.8%) were men and 79/290 (27.2%) were women, resulting in a male-to-female ratio of 2.67 to 1. The main outcomes showed that 53/290 (18.3%) patients died within 28 days, while 73/290 (25.2%) patients died within 90 days of the initial evaluation.

### 3.2. MELD-Na and MELD 3.0 for Predicting 28- and 90-Day Mortality in Whole Sample

ROC analysis was utilized to evaluate the predictive ability of MELD-Na and MELD 3.0 for 28- and 90-day mortality in the entire sample (*n* = 290). The AUC for MELD-Na in predicting 28-day mortality was 0.586 (95% CI: 0.497 to 0.675; *p* = 0.045), while the AUC for MELD 3.0 was 0.614 (95% CI: 0.530 to 0.698; *p* = 0.009). There was no significant difference between the AUCs (*p* = 0.66). The AUC for MELD-Na and MELD 3.0 for 90-day mortality were 0.741 (95% CI: 0.674 to 0.808; *p* < 0.001) and 0.771 (95% CI: 0.707 to 0.836; *p* < 0.001), respectively. There was no significant difference between the AUCs (*p* = 0.60).

### 3.3. ML Model for Predicting 28-Day Mortality

An artificial neural network (ANN)-based machine learning (ML) model was created as previously described. The model was trained on 165 out of 290 patients (56.9%) and tested on the remaining 125 patients (43.1%).

In the training sample, 27 out of 165 patients (16.4%) died within 28 days, while in the testing sample, 26 out of 125 patients (20.8%) died. There was no significant difference between the two groups (X2 = 0.94; df = 1; *p* = 0.333). The training and testing samples had similar 90-day mortality rates, with 39 out of 165 (23.6%) patients and 34 out of 125 (27.2%) patients dying, respectively (X2 = 0.48; df = 1; *p* = 0.489).

**Figure 2:**
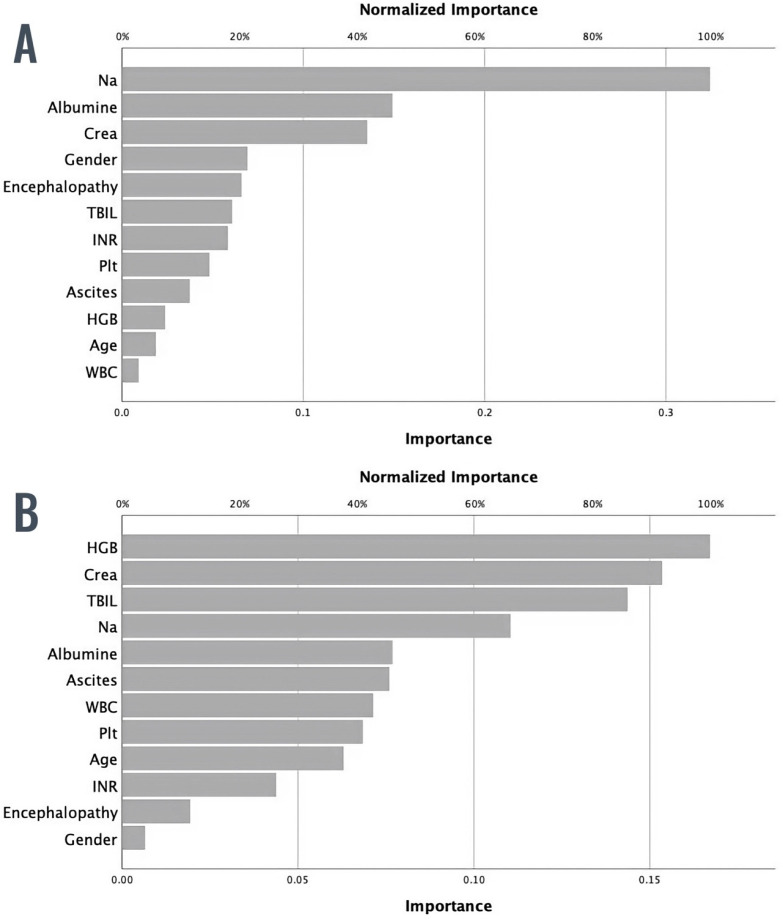
(**A**). Normalized importance of each input variable in ML model for prediction of 28-day mortality in decompensated liver cirrhosis. (**B**). Normalized importance of each input variable in ML model for prediction of 90-day mortality in decompensated liver cirrhosis.

Figure 2A displays the relative importance of each input variable in predicting the outcome, with sodium, albumin, and creatinine being the three most significant variables.

ROC analysis was performed in the validation sample exclusively for the ML model, MELD-Na, and MELD 3.0 scores with 28-day mortality as the outcome. The ML model had an AUC of 0.811 (95% CI: 0.714 to 0.907; *p* < 0.001), while MELD-Na had an AUC of 0.577 (95% CI: 0.435 to 0.720; *p* = 0.226) and MELD 3.0 had an AUC of 0.600 (95% CI: 0.462 to 0.739; *p* = 0.117).

**Figure 3:**
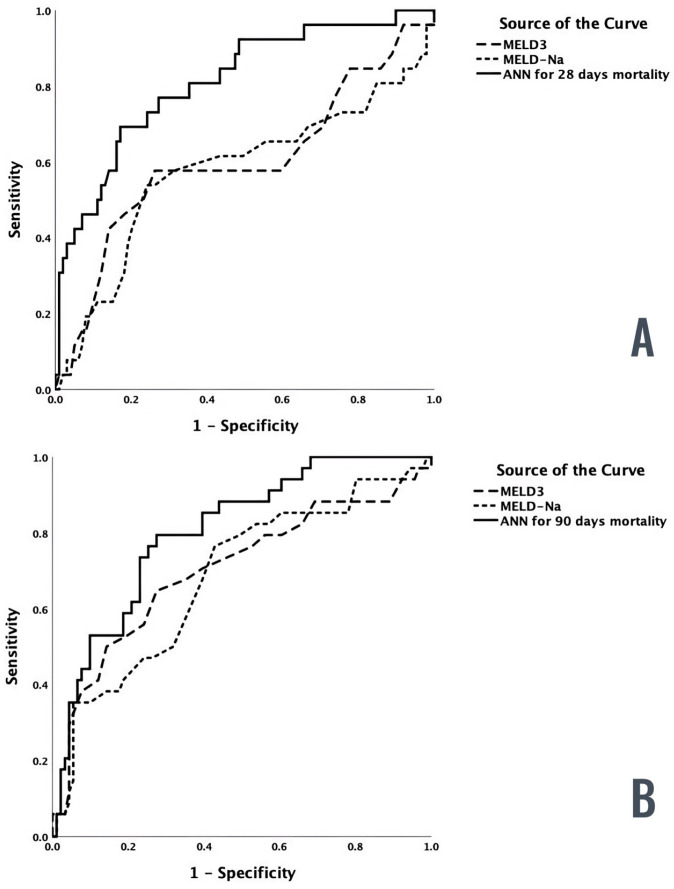
(**A**). Areas under the ROC curve (AUC) for ML model, MELD-Na, and MELD 3.0 scores for prediction of 28-day mortality in decompensated liver cirrhosis. (**B**). Areas under the ROC curve (AUC) for the ML model and MELD-Na scores for prediction of 90-day mortality in decompensated liver cirrhosis.

Statistical analysis revealed that the differences in AUCs between MELD-Na and MELD 3.0, compared to the ML model, were significant (*p* = 0.012 and *p* = 0.005, respectively). Figure 3A shows that the ML model outperformed both MELD-Na and MELD 3.0 in predicting 28-day mortality in decompensated cirrhosis.

### 3.4. ML Model for Predicting 90-Day Mortality

A separate ML model was developed to predict 90-day mortality in patients with decompensated cirrhosis, using the same approach as the 28-day mortality model. Figure 2B presents the normalized importance of each input variable for predicting 90-day mortality, indicating that the three most important input variables were hemoglobin, creatinine, and total bilirubin levels.

ROC analysis was performed in the validation sample for the ML model, MELD-Na, and MELD 3.0 scores, with 90-day mortality as the outcome. The AUC for the ML model was 0.839 (95% CI: 0.776 to 0.884; *p* < 0.001), while MELD-Na had an AUC of 0.682 (95% CI: 0.575 to 0.790; *p* = 0.002) and MELD 3.0 had an AUC of 0.703 (95% CI: 0.590 to 0.816; *p* < 0.001).

When comparing the AUCs of MELD-Na and the ML model, we observed a statistically borderline significant difference (*p* = 0.05). The ML model performed better than the MELD-Na score in predicting 90-day mortality in decompensated cirrhosis (Figure 3B). However, the difference in AUCs between the ML model and MELD 3.0 was not significant (*p* = 0.09).

## 4. Discussion

The objective of this study was to develop and validate machine learning models for predicting short-term mortality (28-day and 90-day) in patients with acute decompensation of liver cirrhosis with mixed etiologies of underlying liver diseases. The models were based on clinical and laboratory parameters obtained at hospital admission, and they demonstrated higher accuracy for predicting short-term mortality when compared with the traditionally used MELD-Na score and the more recently proposed MELD 3.0 score.

Accurate prediction of mortality in patients with decompensated liver cirrhosis is crucial for their management and treatment planning, particularly for timely liver transplantation, given the global organ shortage [28]. Although MELD scores can predict short-term mortality, there have been many attempts to improve this model by adding other parameters of importance, such as sodium, age, bilirubin, and gender, to its formula [8]. Hence, newer derivatives of the MELD score, such as MELD-Na or MELD 3.0 scores, were introduced, which include other relevant parameters in their final formula [7,9].

However, all these attempts are based on conventional statistical methodology, usually based on some form of multivariate regression analysis [29]. In contrast, living organisms and processes within, whether physiological or pathological, can seldom be described with a linear equation. In such circumstances, whereas non-linear, poorly delineated processes exist, the application of ML-based models has repeatedly proven to be superior [30]. As stated in an excellent review by Ghoshal and Das [19], due to the inherent ability of neural network-based systems to identify complex nonlinear interactions, ML-based models are expected to perform better than most linear models such as regression-based models.

In our evaluation of the predictive potential for mortality in patients with decompensated cirrhosis, the corresponding area under the curve (AUC) values for 28- and 90-day mortality were suboptimal for both MELD-Na and MELD 3.0 scores, as shown in Figure 3A,B. Despite the fact that MELD 3.0 includes other relevant parameters such as albumin and female gender to improve the original formula, the improvement between MELD-Na and MELD 3.0 is modest, if not small, as already discussed by the authors of MELD 3.0 [7]. We strongly believe that the problem is not the parameters included in the final formula of the score, but rather the inherent inability of conventional statistical methodology to describe biological systems. Therefore, we speculate that current models used to predict mortality in decompensated liver cirrhosis can be vastly improved with superior predictive methods such as ML.

Several published studies have attempted and succeeded in proving this. For example, Cucchetti et al. [16] demonstrated the clear superiority of an ANN-based model in predicting 90-day mortality of patients with end-stage liver disease compared with the conventional MELD score. More recently, Yu et al. [31] described better predictive performance of an ML model compared to existing scoring systems, including MELD-Na, for predicting 30-day mortality. Finally, a large retrospective cohort study of almost 108,000 US patients with cirrhosis used ML to derive clinical variables used to produce a new score that was more predictive of 1-year mortality than the MELD-Na score [32].

Similarly, the ML models developed for the purpose of this study have demonstrated clear advantages in the short-term prediction of mortality in patients with decompensated cirrhosis compared to standard scoring systems such as MELD-Na or MELD 3.0. In our sample, this superiority was even more emphasized for the prediction of lethal outcomes within 28 days, thus demonstrating the potential of this approach to better identify patients in need of accelerated liver transplantation.

There are certain criticisms that MLs based on ANNs are, in fact, the “black box”. At the most basic level, “black box” means that, for deep neural networks, we do not know how all the individual neurons work together to arrive at the final output. Frequently, we do not understand what happens inside artificial neural networks, what the significance of each particular neuron is, or what the significance of each particular interneuron connection is. But is that not also true for our own nervous system? Yet, we input variables into our brains every day and produce results that are most often actionable. We do not need to know how the car works in order to drive it. The exact way the tool works is not important as long as it provides reliable and accurate predictions [33,34]. This holds true also for ML-based medical decision-making tools, such as in the case of models used in the present study.

We attempted to explain the intricacies of our models, at least partially, with graphs depicting the normalized importance of input variables for each of our particular models (Figure 2A,B). As seen, the most usual suspects, such as sodium, albumin, bilirubin, and creatinine, are among the three most important variables. The hemoglobin level, although not the usual parameter included in the conventional scoring systems for mortality in cirrhosis, has been shown to be a significant risk factor for mortality in liver cirrhosis [35] and emerged as a variable of significance for mortality in other studies that used ML models to predict death in cirrhosis [32].

ML models, which incorporate advanced analytical techniques, are designed with the end-user in mind and aim for ease of implementation in clinical settings. These models can be integrated into existing hospital information systems via software interfaces, allowing for real-time analysis of patient data to generate predictive outputs without the need for manual calculation. Furthermore, unlike traditional scoring systems that rely on static formulas, ML models can continuously learn from new data, potentially offering more accurate and personalized predictions over time. Of course, the deployment of these models in clinical practice would involve initial validation studies followed by training sessions for healthcare providers to ensure familiarity with the system’s functionality and interpretation of its outputs. Such efforts are designed to bridge the gap between advanced machine learning technologies and routine clinical use, ensuring that these tools enhance, rather than complicate, the decision-making process.

It is important to address several limitations of our study. We acknowledge that the predominance of patients with alcoholic liver disease in our sample may limit the generalizability of our findings, as well as the geographic concentration of the data sources. Nevertheless, the clinical course of acute decompensation in cirrhosis is similar, regardless of etiology. While our models show promising results within the studied populations, their performance must be carefully evaluated when extended to other regions. The study is indeed retrospective, but we have invested every effort to thoroughly select patients’ records with complete data on admission, including data on ascites and hepatic encephalopathy grade. We excluded some patients who underwent liver transplantation (Croatian cohort) to harmonize the structure of the investigated cohorts because, in Bosnia, liver transplantations are anecdotal, without a single organ available during the studied period.

Another potential issue is the low predictive accuracy of both MELD-Na and MELD 3.0 scores observed in our study. Yet, this was a retrospective study from real-life cohorts from a certain geographic area, in a population of patients evaluated on admission to the hospital due to decompensation (and not in the general population of cirrhotic patients where most studies evaluating MELD score are conducted), thus reflecting the disease etiology, severity, and clinical patterns of presentation. Some of these factors have been appreciated in the recent analysis, and predictive performances of MELD and its derivates similar to those obtained in our study were reported by other authors as well [36,37].

It is also possible that we could have had even better accuracy of our ML models if we used more clinical variables. However, our intention was to show that ML methodology can yield better results, with practically the same variables commonly used in CP and MELD scores. Of course, ML models can be trained to be more precise with large datasets, preferably from a larger geographical region—the larger, the better. We believe that our sample size was sufficient to demonstrate the clear superiority of ML-based approaches against conventional prognostic scores. It is up to future studies to elaborate this on large datasets and create models that perhaps one day can change the standard of care.

## 5. Conclusions

In conclusion, both ML-based models for predicting short-term mortality (28- and 90-day) in patients with decompensated liver cirrhosis, regardless of the etiology of underlying chronic liver disease, performed better than MELD-Na and MELD 3.0 scores in our validation cohort. Future studies on large datasets are needed to create models that could be used for better allocation of organs and referral of patients for accelerated liver transplantation.

## Figures and Tables

**Table 1:** Baseline characteristics of patients.

Parameter	Characteristics	
*n* = 290	Mean	Standard Deviation
Age (years)	62.97	10.46
WBC (×10^9^/L)	7.67	4.77
Hemoglobin (g/L)	92.34	30.63
Platelets (×10^9^/L)	120	72
Creatinine (μmol/L)	125.10	92.75
Total bilirubin (μmol/L)	121.94	308.89
INR	1.61	0.49
Sodium (mmol/L)	135	6
Albumin (g/L)	26.81	5.87
MELD-Na	18	8
MELD 3.0	21	8
	***n***	**%**
**Ascites**		
Grade 0	69	23.8
Grade I	186	64.1
Grade II	35	12.1
**Encephalopathy**		
Grade 0	191	65.9
Grade I	15	5.2
Grade II	47	16.2
Grade III	36	12.4
Grade IV	1	0.3
**Etiology of cirrhosis**		
Alcohol	230	79.3
HBV/HCV	36	12.4
Other + cryptogenic *	12	4.1
PBC/PSC	7	2.4
NAFLD	5	1.7

* Other etiologies, such as autoimmune hepatitis, non-alcoholic steatohepatitis, Wilson’s disease, and cryptogenic cirrhosis.

## Data Availability

The data presented in this study are available on request from the corresponding author.
